# Dual role of macrophage migration inhibitory factor (MIF) in human breast cancer

**DOI:** 10.1186/1471-2407-9-230

**Published:** 2009-07-14

**Authors:** Eva Verjans, Erik Noetzel, Nuran Bektas, Anke K Schütz, Hongqi Lue, Birgitt Lennartz, Arndt Hartmann, Edgar Dahl, Jürgen Bernhagen

**Affiliations:** 1 Department of Biochemistry and Molecular Cell Biology, RWTH Aachen University Hospital, Aachen, Germany; 2 Molecular Oncology Group, Institute of Pathology, RWTH Aachen University Hospital, Aachen, Germany; 3 Institute of Pathology, University of Erlangen, Erlangen, Germany

## Abstract

**Background:**

Macrophage migration inhibitory factor (MIF) is a pleiotropic cytokine and mediator of acute and chronic inflammatory diseases. MIF is overexpressed in various tumours and has been suggested as a molecular link between chronic inflammation and cancer. MIF overexpression is observed in breast cancer but its causal role in the development of this tumour entity is unclear.

**Methods:**

MIF levels in breast cancer cell lines were determined by ELISA and Western blot. CD74 was measured by Western blot, fluorescence microscopy and flow cytometry. Cell proliferation was studied by BrdU incorporation, cell adhesion by Matrigel adhesion assay, and cell invasion by migration assay through Matrigel-coated filters using the Transwell system. MIF expression in primary human breast cancers was measured by tissue microarray and a semi-quantitative immunoreactivity score (IRS) and comparison with histopathological parameters and patient outcome data.

**Results:**

MIF was abundantly expressed in the non-invasive breast cancer cell lines MDA-MB-468 and ZR-75-1, but not in invasive MDA-MB-231 cells, which in turn expressed higher levels of the MIF-receptor CD74. Stimulation with exogenous MIF led to a dramatic upregulation of MIF secretion (50-fold) in MDA-MB-231 cells. Autocrine MIF promoted tumour cell proliferation, as indicated by blockade of MIF or CD74 in MDA-MB-231 and MDA-MB-468, and MDA-MB-231 invasiveness was enhanced by exogenous MIF. We correlated the expression of MIF with histopathological parameters and patient outcome data, using a tissue microarray of 175 primary invasive breast cancers and 35 normal control tissues. MIF was upregulated in breast cancer versus normal tissue (median IRS = 8 versus 6). MIF expression showed positive correlations with progesterone (p = 0.006) and estrogen (p = 0.028) receptor expression, markers of a favourable prognosis and a negative correlation to tumour size (p = 0.007). In line with these data, disease-specific overall (OS) as well as recurrence-free (RFS) survival was significantly improved in breast cancer patients with abundant cytosolic MIF expression compared to MIF low expressers (5-year OS = 67% versus 50%, p = 0.0019; 5-year RFS = 52% versus 36%, p = 0.0327).

**Conclusion:**

We conclude that intracellular expression of MIF in breast cancer cells is beneficial, whereas extracellular MIF may play a pro-oncogenic role in promoting breast cancer cell-stroma interactions.

## Background

Macrophage migration inhibitory factor (MIF) is a pleiotropic cytokine and upstream regulator of the host immunity that promotes cellular inflammatory responses such as mitogen-activated protein kinase (MAPK) signalling, tumour necrosis factor-α (TNF-α) secretion or cyclooxygenase-2 (COX-2) activity. Owing to its inflammatory activities, MIF is a pivotal mediator of acute and chronic inflammatory diseases including septic shock, rheumatoid arthritis, and atherosclerosis [1-4]. MIF is not only secreted by immune cells, but also by parenchymal and tumour cells upon inflammatory and stress stimulation [1]. Sharing an architectural 3D similarity with the atherogenic and angiogenic chemokine interleukin-8 (IL-8)/CXCL8, MIF was found to function as a non-cognate ligand of CXCR2 and as chemokine-like function (CLF) chemokine. Inflammatory leukocyte recruitment is dependent on MIF-CXCR2 and MIF-CXCR4 interactions [5]. As observed for CLF chemokines, MIF action is not limited to the extracellular space, but also occurs intracellularly. MIF is found in the cytosol of various cell types, where it contributes to cell survival, cell cycle and homeostasis control. Intracellular MIF activities are linked to c-Jun activation domain binding protein-1 (JAB1), the tumour suppressor protein p53, and the thiolprotein oxidoreductase (TPOR) activity of MIF [1,6-8].

MIF has been implicated in cancerogenesis already as early as in 1999, when Mitchell and colleagues found that it mimics the action of oncogenic RAS protein by inducing sustained ERK1/2 signalling [9]. Meanwhile, it has been appreciated that MIF constitutes an important link between chronic inflammation and cancer. Of note, MIF levels are markedly elevated in numerous tumour entities such as prostate tumours, breast cancer, or colon carcinomas [10-12]. Recombinant MIF (rMIF) promotes cell proliferation and migration and blockade of MIF by antibodies or gene deletion leads to reduced proliferation and inhibition of tumour growth and angiogenesis [13-18]. Pro-tumourigenic activities of MIF involve the MIF receptor CD74 and stimulation of the phosphoinositide-3-kinase (PI3K)/AKT/SRC signal transduction cascade [9,19-21]. Moreover, MIF inhibits p53-dependent gene expression and suppresses apoptosis. Secretion of MIF by tumour cells has been proposed to enhance tumour cell proliferation by autocrine amplification as known for other growth factors expressed by cancer cells [6,18,22,23]. CD74 is expressed on various cancer cells, i.e. prostate cancer cells, B lymphomas, or gastric carcinomas [24-26], but its expression in breast cancer has not been studied. Of note, binding of MIF to CD74 leads to the recruitment of the hyaluronate receptor CD44 and CD74/CD44 complexes have been implicated in pro-tumourigenic MIF signalling processes [27,28]. Although its precise role has remained unclear, CD44 has been amply associated with breast cancer pathogenesis [29,30].

MIF is overexpressed in various breast cancer cell lines and human breast cancer tissue but its functional role in the pathogenesis of this tumour entity is poorly understood [12,21,31]. Given that intracellular MIF has been reported to have a beneficial role in improving cell homeostasis and that secreted and extracellular MIF has various pro-tumourigenic and pro-inflammatory effects, it is conceivable that the clinicopathological role of MIF in breast cancer is complex and may vary with tumour stage and type. Hagemann *et al. *investigated MIF induction in breast tumour-stroma interactions. MIF was identified as a major gene product upregulated in breast cancer cells upon coculture with macrophages. TNFα-triggered tumour cell-derived MIF led to increased macrophage metalloproteinase production rates and facilitated tumour cell invasion [32].

To clarify the causal involvement of MIF in breast cancer, we investigated the role of MIF and its receptor CD74 in cytokine production, proliferation, and invasion of invasive versus non-invasive breast cancer cells. Moreover, we undertook a comprehensive study correlating MIF expression levels in 175 breast cancer specimens with clinicopathological parameters and patient outcome data including overall (OS) and recurrence-free survival (RFS).

## Methods

### Breast cancer tissue microarray (TMA)

A TMA was constructed as described previously [33] and contained 289 non-selected formalin-fixed, paraffin-embedded primary breast cancers (stage I-IIIC) together with matched normal breast tissue. All patients gave informed consent for retention and analysis of their tissue for research purposes and the study was approved by the Ethical Committee of the University of Regensburg. An experienced surgical pathologist (A.H.) evaluated H&E-stained slides of all specimens before construction of the TMA to identify representative tumour areas and to re-evaluate tumour grading. Clinical follow-up, provided by the Central Tumor Registry Regensburg, Germany, was available for all breast cancer patients with a median follow-up period of 79 months (0–148 months). Clinicopathologic parameters of breast cancer cases included in the TMA are summarized in a supplementary table (see Additional File 1).

### Cells and reagents

Cell culture reagents were purchased from Invitrogen. Neutralising anti-CD74 monoclonal antibodies (mAbs) (sc-5438) were from Santa Cruz and FITC-labelled anti-CD74 was from Biomedia. The anti-MIF mAb NIH/III.D9 was from the Bucala lab (New Haven, USA) and control IgG was of the IgG1 subtype [5]. Anti-actin C4 mouse mAb was from MP Biomedicals, sheep anti-mouse (Fab')_2 _from GE Healthcare and FITC-labelled anti-mouse antibody from Dianova. Polyclonal anti-MIF antibody (Ka565) and rMIF were generated as described [5].

The normal breast epithelial line MCF-12A, non-invasive breast cancer cell lines MDA-MB-468 (infiltrating adenocarcinoma) and ZR-75-1 (infiltrating ductal carcinoma), and highly invasive MDA-MB-231 cells (invasive ductal carcinoma) were cultured as described [21].

### Cell lysis and Western blotting analysis

Cell lysates for Western blotting were prepared from 100.000 cells and subjected to NuPAGE^® ^electrophoresis/Western blotting as described [21]. MIF and CD74 were detected by anti-CD74 (sc-5438; Santa Cruz) and anti-MIF (Ka565) primary antibodies, respectively; anti-actin (C4; 1:500 dilution) was used for standardisation. Following treatment with HRP-conjugated secondary Ab, bands were quantified by ECL chemiluminescence as described [21]. Quantifications of blots are representative of 3–5 independent experiments.

### Stimulations with rMIF and MIF ELISA

Cells were incubated at 100,000 cells per well in medium containing 10% FCS for 24 h at 37°C. Medium was adjusted to low serum (0.5% FCS) and cells cultured for 48 h. 150 ng/ml of rMIF were added to cells 10 min to 48 h before the end of the incubation. Control incubations were performed for each time interval. Supernatants were analysed for MIF by commercial human MIF ELISA (R&D Systems). For each time interval, 3–6 independent experiments were performed and each sample was analysed by triplicate ELISA measurements.

### Fluorescence microscopy

Living cells were seeded on cover slips in 6-well plates. Washed cells were incubated in PBS with FITC-labelled anti-CD74 Ab for 1 h. Control cells were treated with an irrelevant FITC-labelled antibody. After fixation, cells were analysed by fluorescence microscopy and 10 pictures evaluated for each sample.

### Proliferation assay

8,000 cells were plated and proliferation measured over 24–72 h followed by a 6 h incubation with BrdU using a commercial BrdU proliferation kit from Roche Diagnostics. Antibodies for neutralisation experiments and isotype control IgG (58 μg/ml), and/or rMIF at different concentrations (0, 10, 50, and 150 ng/ml) were added at the beginning of the incubation period.

### Matrigel invasion assay

Matrigel invasion assay was conducted in 24-well plates applying the Transwell device containing microporous 8 μm membranes (Corning, USA). Membranes were coated with Matrigel (500 ng/ml) and MDA-MB-231 cells seeded in the upper chamber containing basal medium with 0.5% BSA. rMIF was added to the lower chamber at final concentrations between 0 and 100 ng/ml. 10% FCS served as positive chemoattractant control. Migration/invasion was followed for 72 h. Transwell inserts were transferred to a new plate and cells adhering to the lower surface were stained with 8 mM calcein (Calbiochem). The total number of invading cells was acquired in six representative fields using fluorescence microscopy (10 × magnification).

### Real-time PCR

Total RNA processing and real-time PCR were performed as described [34]. PCR primer sequences: MIF-F312 (5'AGAACCGCTCCTACAGCAAG 3') and MIF-R432 (5' GAGTTGTTCCAGCCCACATT 3'). Intensity was normalised to GAPDH. PCR conditions were: 95°C for 5 min; 40 cycles of 95°C for 20 s, 61°C for 61 s, and 72°C for 12 s; 60 s at 72°C. Relative quantification of gene expression was performed using the comparative CT (ΔΔ CT) method.

### Immunohistochemistry

Three μm micrometer sections were prepared and immunohistochemically stained as described elsewhere [34]. Tissue sections were stained with an anti-human MIF antibody (MAB289, R&D Systems) at a 1:400 dilution.

### Statistical analyses

*In vitro *assays: Results are expressed as means ± SD. Graphs were created with Origin Pro 7.0 (Origin Corp.) and data analysed with Student's t-test.

TMA: MIF protein expression was quantified using the immunoreactive score of Remmele and Stegner which consists of the mathematical product of quantity (score 0–4) and intensity (score 1–3) of protein staining [35]. Statistical analyses were performed using SPSS version 14.0 (SPSS). Two-sided Tarone-Ware tests/Fisher's exact tests were performed to correlate RFS and OS with MIF expression and clinicopathological parameters. Multivariate proportional hazard Cox regression was performed to test for independent prognostic relevance. The limit for reverse selection procedures was p = 0.2. The proportionality assumption for all variables was assessed with log-negative-log survival distribution functions.

Differences were considered significant at p values < 0.05.

## Results

### Differential expression of MIF and CD74 in invasive versus non-invasive breast cancer cells

**Figure 1:**
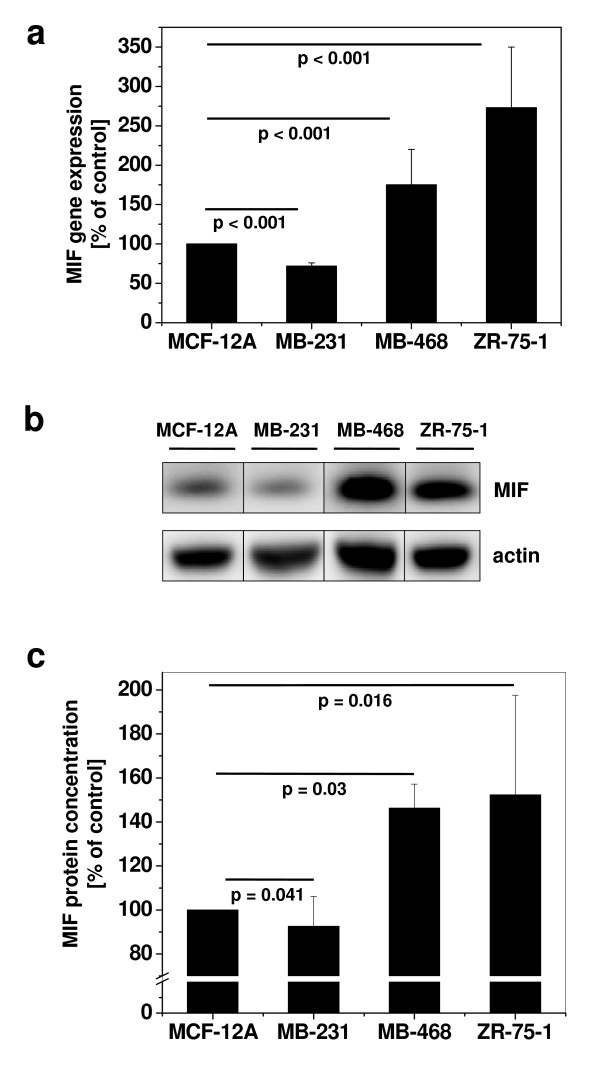
**MIF is overexpressed in breast cancer cells, but differs in its expression between invasive versus non-invasive cells**. **(a) **Comparison of MIF mRNA levels in non-tumorous MCF-12A cells with the invasive MDA-MB-231 and non-invasive MDA-MB-468 and ZR-75-1 breast cancer cell lines. mRNA of non-stimulated cells was isolated, transcribed to cDNA, and MIF gene expression measured by real-time PCR. Gene expression levels are shown relative to the expression level in MCF-12A. Data are means of two determinations and are representative of two independent experiments. **(b) **As in **(a) **but comparison of MIF protein levels by Western blot. Actin was used as a loading control. **(c) **Quantification of Western blot by densitometry, using actin for standardization. The quantification data are means ± SD of 4 independent experiments. P values indicate statistically significant differences between MIF expression in the breast cancer cell lines compared to MCF-12A.

We first applied quantitative PCR (Figure 1a) and Western blotting (Figure 1b–c) to study the mRNA and protein expression levels of MIF, respectively, in various invasive and non-invasive ductal breast cancer cell lines in comparison to normal epithelial cells. Non-invasive MDA-MB-468 and ZR-75-1 cells showed an upregulation of MIF compared to benign epithelial MCF-12A cells, both at the mRNA and protein level, confirming earlier observations [21]. Surprisingly, invasive MDA-MB-231 breast cancer cells exhibited significantly lower MIF mRNA and protein levels when compared with the non-invasive cells or MCF-12A. Thus, MIF *de novo *synthesis and intracellular protein storage differ between normal epithelial and breast cancer cells and highly invasive cells produce low concentrations of this cytokine.

**Figure 2:**
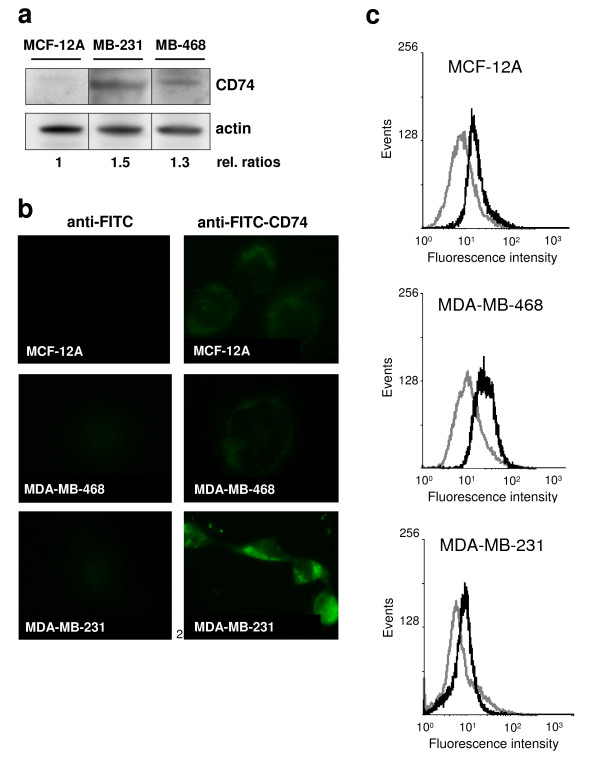
**The MIF receptor CD74 is overexpressed in invasive MDA-MB-231 cells**. **(a) **Comparison of CD74 expression levels between MDA-MB-231, non-invasive MDA-MB-468, and non-tumorous MCF-12A cells by Western blot analysis. Lysates of non-stimulated cells were analysed by Western blot and band densitometry analysis against human CD74 and actin. Numbers indicate relative CD74/actin ratios. The quantification is representative of three independent experiments. **(b) **Comparison of CD74 expression levels between MDA-MB-231, non-invasive MDA-MB-468, and non-tumorous MCF-12A cells by fluorescence microscopy. Surface-expressed CD74 was revealed by FITC-labelled anti-CD 74 Ab. As a negative control, cells were labelled with a FITC-labelled secondary anti-mouse antibody. Photographs are representative of two independent experiments.

Next, we measured expression of the MIF receptor CD74 by Western blotting, immunocytochemistry (IHC), and flow cytometry (Figure 2). Generally, CD74 expression was not very prominent in the cells tested. Western blotting and IHC analysis showed marked expression of CD74 in MDA-MB-231, but expression was weak to absent in MDA-MB468 and MCF-12A, as judged by these methods, respectively. IHC indicated that CD74 expression in MDA-MB-231 was mostly intracellular, an observation that was in line with previous knowledge on the cellular distribution of CD74/Ii. Flow cytometry analysis confirmed that in MDA-MB-231 cells, some portion of CD74 was localised on the cell surface (Figure 2c). Interestingly, this method also revealed surface expression of CD74 on MDA-MB-468 and MCF-12A cells, which was comparable to that on MDA-MB-231 cells. Overall, invasive MDA-MB-231 cells exhibited the highest levels of CD74, which were mostly intracellular. Thus, CD74 appears to be primarily expressed in invasive breast cancer cells, indicating that such tumour cells may be prone to stimulation with MIF.

### MIF secretion by breast cancer cells is dramatically upregulated by exogenous MIF

**Figure 3:**
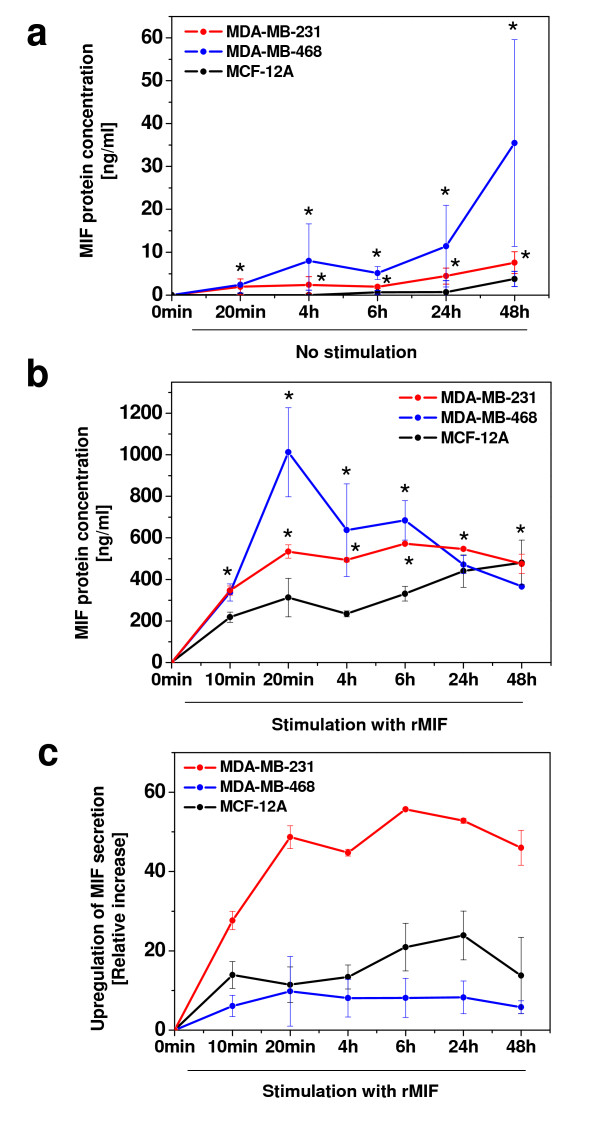
**MIF secretion from breast cancer cells is upregulated by exogenous MIF**. **(a) **Comparison of MIF secretion levels in unstimulated non-invasive MDA-MB-468, invasive MDA-MB-231 tumour cells, and MCF-12A control cells. Supernatants of 100,000 cells were subjected to human MIF ELISA at the indicated time intervals. Of note, MDA-MB-468 cells secreted MIF levels of up to 35 ng/ml. Asterisks indicate statistical significance compared to MCF-12A. **(b) **Exogenous rMIF dramatically upregulates the secretion of MIF from breast cancer cells. As in **(a)**, but stimulation of cells with 150 ng/ml rMIF for the indicated time periods. Asterisks indicate statistical significance compared to MCF-12A. **(c) **Invasive MDA-MB-231 breast cancer cells exhibit the highest relative increase in MIF secretion. Relative increase of MIF secretion calculated from **(b)**. To account for the addition of the 150 ng/ml exogenously added rMIF, this value was subtracted for all incubations. Data represent means ± SD of three determinations and two **(b) **or three **(a and c) **independent experiments.

Upon inflammatory stimulation, tumour cells secrete growth factors and cytokines, which amplify neoplastic tumour cell transformation and participate in tumour-stroma interactions and activation of tumour-associated macrophages (TAMs). MIF is produced and secreted by both monocytes/macrophages and tumour cells and has pro-tumourigenic activities *in vitro*. To begin to study potential amplifying effects of exogenous MIF, i.e. MIF released in the tumour microenvironment by TAMs or breast cancer cells themselves, we examined the secretion of MIF in unstimulated breast cancer cells and in those exposed to exogenous recombinant MIF (rMIF). First, spontaneously secreted MIF levels were measured in unstimulated MCF-12A, MDA-MB-468, and MDA-MB-231 cells by ELISA. In both tumour cell lines, accumulated MIF concentrations in the supernatants reached appreciable concentrations (Figure 3a), whereas no constitutive MIF release was observed in MCF-12A. The MIF secretion rate in MDA-MB-468 was 4–6-fold higher than in MDA-MB-231, reaching >35 ng/ml after 48 h. Thus, in cycling breast cancer cells, MIF production and secretion rates exceeded re-endocytosis and degradation.

We next studied the effect of rMIF on the secretion rate of endogenous MIF. Notably, treatment with 150 ng/ml rMIF not only stimulated endogenous MIF secretion constituting an autocrine loop, but led to a remarkable upregulation of MIF secretion in both non-invasive and invasive tumour cells (Figure 3b). MIF levels were way in excess over the added 150 ng/ml and significantly increased over the release seen in MCF-12A. The most dramatic effect was seen in MDA-MB-468 stimulated with rMIF for 20 min, where secreted MIF levels reached up to 1000 ng/ml, but also in MDA-MB-231, in which MIF secretion reached 500 ng/ml. Upregulation of MIF secretion by MIF was specific as boiled rMIF and a control buffer had no effect (data not shown). Whereas in MDA-MB-468, secreted MIF peaked at 20 min and then declined, secretion in MDA-MB-231 exhibited a broad plateau between 20 min and 48 h of stimulation with rMIF (Figure 3b). We calculated the relative upregulation factors of MIF secretion (ratio of rMIF-induced MIF secretion over basal MIF secretion) in both breast cancer cell types. This analysis demonstrated that the invasive cells exhibited dramatic relative upregulation factors of up to 55-fold (Figure 3c), whereas relative upregulation in MDA-MB-468 was less than 10-fold and even lower than in normal breast epithelial cells. rMIF-triggered secretion of endogenous breast cancer cell-MIF is likely to encompass both a burst of preformed MIF release at 10–20 min and *de novo *synthesized MIF, appearing 4 h after inflammatory stimulation with rMIF. Thus, MIF secretion from breast cancer cells could be strongly influenced by auto- or paracrine MIF effects in the tumour-stroma microenvironment.

### MIF supports the proliferation rate of breast cancer cells

**Figure 4:**
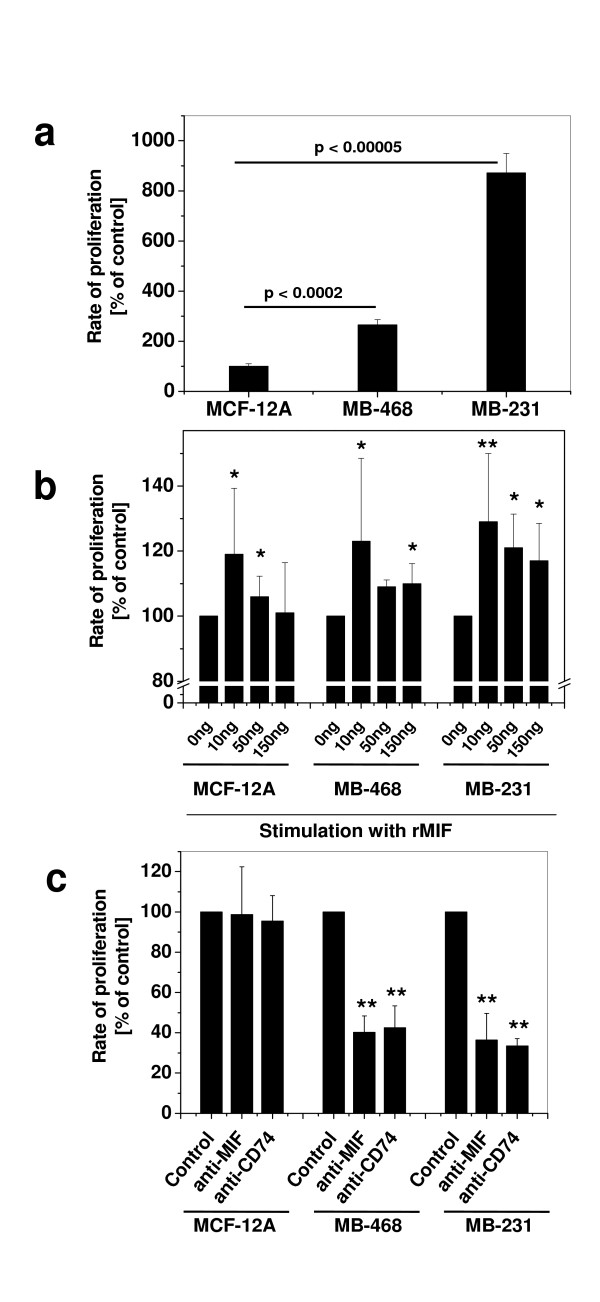
**MIF augments the proliferation rate of breast cancer cells**. **(a) **Proliferation of unstimulated breast epithelial and breast carcinoma cell lines. Proliferation was measured by BrdU assay and is represented as percent of the baseline proliferation rate of MCF-12A. Proliferation rates of MDA-MB-231 and -468 are markedly and significantly (p values indicated) higher than that of MCF-12A. **(b) **Exogenous rMIF slightly but significantly enhances the proliferation rate of normal breast epithelial and breast carcinoma cell lines. As in **(a) **except that rMIF at indicated concentrations was added to cells for 24 h. Control cells (0 ng/ml) received control buffer (final dialysis refolding buffer). Asterisks indicate statistically significant differences compared to the corresponding control incubations: *, p < 0.05; **, p < 0.01. **(c) **Endogenous MIF supports proliferation of breast cancer cells by an autocrine loop and inhibition of proliferation by anti-MIF and anti-CD74 antibodies. Unstimulated proliferation of breast cancer cells as in **(a) **was compared to that of MCF-12A in the presence versus absence of neutralising anti-MIF and anti-CD74 antibodies. Control cells were incubated with equivalent amounts of PBS and isotype IgG had no effect (data not shown). Statistically significant differences (in comparison to cells not treated with antibody) are indicated by asterisks: *, p < 0.05; **, p < 0.01. Proliferation rates are means ± SD of two **(b) **or three **(a, c) **independent experiments with two **(a) **or three **(b, c) **determinations.

We next compared unstimulated proliferation rates in two breast cancer cell lines and MCF-12A by BrdU incorporation assay. Invasive MDA-MB-231 cells showed the highest baseline proliferation rate (~7.8-fold), while MDA-MB-468 proliferated 2.1-fold stronger than MCF-12A cells (Figure 4a). We then stimulated the cells with different rMIF concentrations, ranging from 0–150 ng/ml. rMIF enhanced the proliferation of both tumour cell lines and also that of MCF-12A (Figure 4b). Peak stimulation was obtained with 10 ng/ml rMIF, a typical concentration measured in (patho)physiological fluids [2], but overall, proliferation rates induced by rMIF were moderate (≤ 1.3-fold). To test for autocrine effects, cells were cultured under unstimulated conditions and endogenously produced MIF blocked by neutralizing anti-MIF antibody or by an antibody against CD74. Both antibodies, but not an isotype IgG, led to markedly reduced proliferation rates in MDA-MB-468 and MDA-MB-231 by 50–70%. By contrast, proliferation of MCF-12A was not affected (Figure 4c). The remaining proliferation rates of 30–50% imply engagement of additional MIF signalling pathways such as the MIF/CXCR4 pathway [5].

In conjunction, these experiments showed that proliferation of both non-invasive and invasive breast cancer cells is driven by autocrine MIF action, encompassing the secretion of endogenous MIF and signalling through MIF/CD74. It is of note that the breast cancer cell type exhibiting the highest overall (surface + endolysosomal) CD74 expression levels (see Figure 2), showed the greatest response to MIF.

### MIF promotes migration and invasion of breast cancer cells

**Figure 5:**
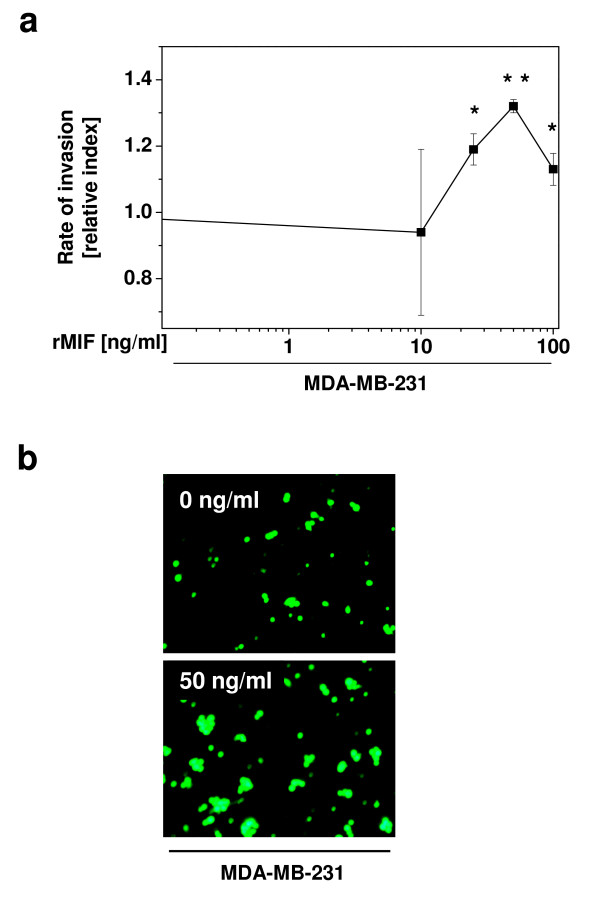
**MIF promotes the migration and invasion of breast cancer cells**. Invasive MDA-MB-231 cells were incubated in the upper Matrigel-coated insert of a Transwell chamber and their migration and invasion measured in response to rMIF added as a chemoattractant to the lower chamber at indicated concentrations. Invaded Calcein-labelled cells were counted by fluorescence microscopy. **(a) **Quantification of 5 independent experiments taking 10 pictures each. Data points are means ± SD and asterisks indicate significant increases of the invasion rate (*, p < 0.05; **, p < 0.01). **(b) **Representative fluorescence microscopy images comparing the effect 50 ng/ml MIF with a control incubation (0 ng/ml MIF).

MIF has been demonstrated to directly and indirectly act as a chemoattractant for leukocytes, fibroblasts, and tumour cells [5,36,37]. To study the pro-invasive activities of MIF on breast cancer cell migration, MDA-MB-231 cells were calcein-labelled and subjected to Transwell assays applying Matrigel-coated filters. MIF significantly and dose-dependently stimulated the migration/invasion of MDA-MB-231 (Figure 5; maximum effect at 50 ng/ml rMIF). Thus, rMIF promotes the migration and invasion of breast cancer cells through basement membrane-like layers in a chemokine-like manner, confirming the notion that MIF produced in the microenvironment surrounding breast tumour cells may act in a pro-invasive manner.

### MIF is overexpressed in breast cancer tissue in vivo

**Figure 6:**
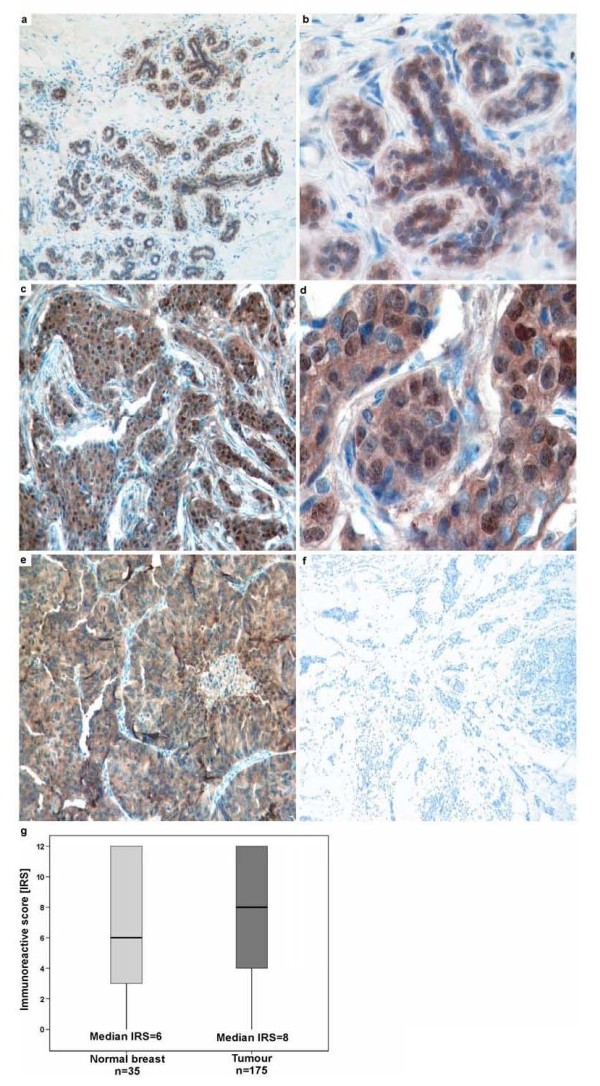
**MIF is overexpressed in breast cancer tissue**. **(a-f) **Immunohistochemical stainings of MIF protein expression on a tissue microarray (TMA) of human breast cancer tissue specimens. Representative stainings applying a MIF antibody are shown. **(a, b) **Normal breast epithelia with moderate MIF protein expression (IRS = 4). **(c, d) **Invasive breast tumour with strong MIF protein expression (IRS = 8). **(e, f) **Samples of invasive mamma carcinoma were used as positive and negative control. For negative control, the primary antibody was omitted. Original magnifications: 100× for a, c, e, f; 400× for b, d. **(g) **Box plot analysis illustrating the distribution of MIF protein expression in normal breast tissue and breast cancer tissue. The y axis indicates the IRS of cytoplasmic MIF protein staining in normal (n = 35) and breast cancer tissue (n = 175) analysed on the TMA. Median MIF expression was found to be increased in human breast tumours (median IRS = 8) compared to normal breast epithelia (median IRS = 6). Horizontal lines: group medians; boxes: 25–75% quartiles; vertical lines: range and minimum.

The *in vitro *studies suggested that exogenous MIF derived from the tumour microenvironment promotes proliferation, adhesion, and invasion of CD74^+ ^invasive breast cancer cells. However, the association between MIF serum levels or MIF expression within breast cancer tissue and breast cancer progression in humans has been controversial. We thus performed a comprehensive retrospective study correlating MIF expression levels with clinical and pathological markers relevant in breast cancer using a tissue microarray (TMA) with 175 primary invasive breast cancers and 35 normal breast tissues. For patient characteristics see supplementary table (see Additional File 1). MIF protein expression was observed in normal breast epithelial cells (Figure 6a, b) and breast tumour cells (Figure 6c–e). Although MIF expression was variable in both normal and malignant breast tissue, abundant MIF expression was predominantly found in tumour cells. Overall, we found that MIF levels were upregulated in breast cancer tissue compared to normal breast epithelium according a semi-quantitative immunoreactivity score (Remmele and Stegner; Figure 6g).

### High MIF expression in breast tumour tissue correlates with tumour size and hormone receptor status and is associated with favourable survival

Next, we correlated MIF expression levels in primary human breast cancer with clinicopathological parameters. Table 1 shows the correlations obtained when high (IRS = 6–12) and low (IRS = 0–4) MIF expression was compared by descriptive Fisher's exact test with tumour size, lymph node status [38], histological grade, as well as estrogen (ER), progesterone (PR), and epidermal growth factor (EGFR) receptor status. MIF expression was highly significantly associated with tumour size (pT) in an inverse manner (p = 0.007). Thus large tumours (>2 cm) being associated with poor prognosis predominantly expressed low levels of MIF (IRS<4). A highly significant positive correlation was found between abundant MIF expression and PR status (p = 0.006). In addition, positive correlations with the ER and EGFR status were measured (p = 0.028 and p = 0.038, respectively). Thus surprisingly, abundant MIF expression in breast tumour tissue correlates with markers associated with a favourable prognosis, i.e. pT, positive ER and PR status. No correlations were observed with lymph node status and histological grade.

**Table 1:** Clinicopathological parameters in relation to MIF protein expression

	MIF protein expression			
	
Variable	n^a^	IRS (0–4)	IRS (6–12)	P value^d^
Tumour size^b^				
pT1	48	13	35	**0.007**
pT2	86	24	62	
pT3	12	3	9	
pT4	27	16	11	
Lymph node status^b^				
pN0	67	17	50	0.190
pN1-3	100	35	65	
Histological grade				
G1	19	9	10	0.933
G2	73	18	55	
G3	79	29	50	
				
Histological type				
invasive ductal	145	51	94	0.496
invasive lobular	13	3	10	
other	17	5	12	
				
Estrogen receptor status				
negative (IRS^c ^0–2)	46	21	25	**0.028**
positive (IRS 3–12)	96	26	70	
Progesterone receptor status				
negative (IRS 0–2)	101	39	62	**0.006**
positive (IRS 3–12)	49	8	41	
EGFR status				
negative (IRS 0)	65	28	37	**0.038**
positive (IRS 1–3)	92	25	67	

^a ^Only female patients with primary, unilateral, invasive breast cancer were included. Significant p values are marked in bold face. ^b ^According to the TNM classification of Sobin and Wittekind [38]. ^c ^IRS = immunoreactive score according to Remmele and Stegner [35]. ^d ^Fisher's exact test. Significant p values are marked in bold face.

**Figure 7:**
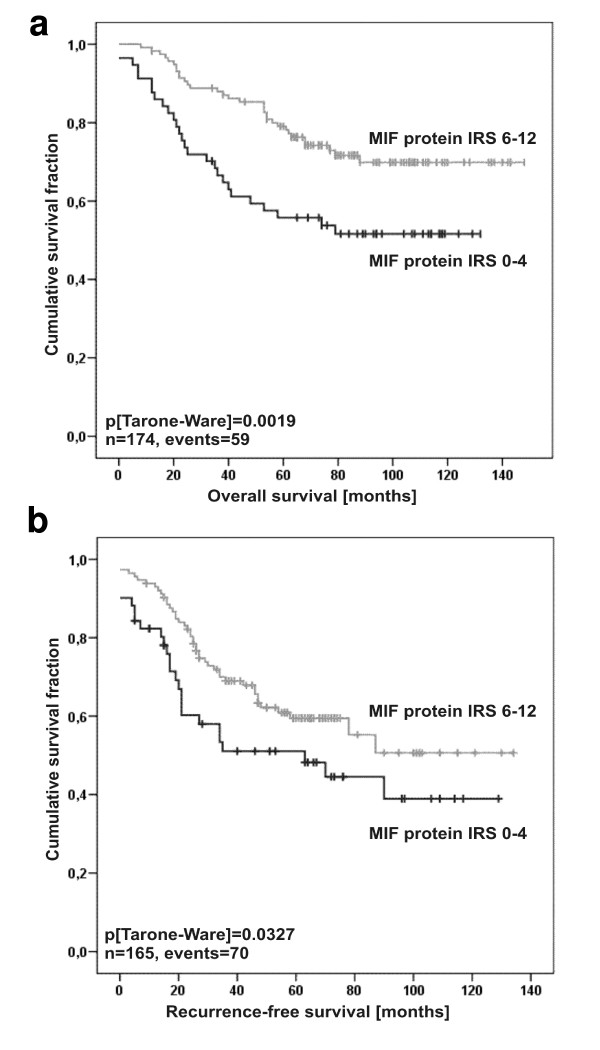
**High MIF expression in breast tumour tissue positively correlates with survival**. Kaplan-Meier analyses of overall survival (OS) **(a) **and recurrence-free survival (RFS) **(b) **in relation to MIFprotein expression in breast cancer tissue specimens of 174 and 165 patients, respectively. Distribution of time (months)- and tumour-related death among 174 breast cancer patients with abundant (upper graph) or low (lower graph) MIF expression is shown. **(a) **Patients featuring low MIF protein expression (IRS = 0–4) have a reduced estimated mean five-year OS survival rate of 50% compared to 67% survival probability for patients with strong MIF expression (IRS = 6–12). **(b) **Patients exhibiting low MIF protein expression (IRS = 0–4) have an increased risk for tumour relapse (mean RFS rate of 52%) after five years compared to 36% for patients with strong MIF expression (IRS = 6–12).

The obtained correlation between MIF expression in breast tumour tissue and tumour size and hormone receptor levels tempted us to postulate that MIF overexpression might be correlated with increased tumour-specific survival in breast cancer patients. Patients with strong cytosolic MIF expression in the breast cancer tissue (IRS = 6–12) showed a highly significant increase in overall survival (OS) compared to the MIF low expresser group with an IRS = 0–4 (5-year OS = 67% versus 50%, respectively; p = 0.0019; Figure 7 and Table 2). In line with these findings, 5-year recurrence-free survival (RFS) was 52% in the MIF high expressers compared to 36% in the low expressers (p = 0.0327; Figure 7 and Table 2). IRS analysis of the PR status paralleled that of MIF with high PR concentrations correlating with increased OS and RFS rates (p < 0.001 and p = 0.01, respectively). Of note, tumour size, lymph node status, and histological grade positively and significantly correlated with OS and RFS in our cohort of breast cancer patients underscoring the suitability of this cohort to analyse new prognostic marker molecules. Multivariate Cox regression models including factors possibly influencing OS/RFS in relation to MIF protein expression failed to exhibit significance, indicating that MIF is not an independent prognostic marker in breast cancer, probably due to its close relation to hormone receptor status (data not shown).

**Table 2:** Univariate analysis of clinicopathological parameters influencing recurrence-free survival (RFS) and overall survival (OS).

Variable	RFS			OS		
	n^a^	events	p value^d^	n	events	p value
***Clinicopathological factors:***						
Tumour size^b^						
pT1	46	10		48	8	
pT2	83	39		86	30	
pT3	11	5	**< 0.001**	12	3	**0.001**
pT4	24	16		27	18	
Lymph node status^b^						
pN0	66	14		68	11	
pN1-3	97	53	**< 0.001**	101	44	**< 0.001**
Histological grade						
G1	18	4		19	5	
G2	69	23		74	18	
G3	78	43	**< 0.001**	80	37	**0.003**
Histological type						
invasive ductal	138	63		142	50	
invasive lobular	12	3		14	5	
other	15	4	0.167	17	5	0.876
Estrogen receptor status						
negative (IRS^c ^0–2)	46	24		46	19	
positive (IRS 3–12)	93	32	0.065	98	28	0.076
Progesterone receptor status						
negative (IRS^c ^0–2)	95	48		102	44	
positive (IRS 3–12)	50	13	**0.010**	50	8	**< 0.001**
MIF protein expression						
weak (IRS^c ^0–4)	51	26		57	27	
strong (IRS 5–12)	114	44	**0.033**	117	32	**0.002**
EGFR status						
negative (IRS^c ^0)	59	21		65	23	
positive (IRS 1–3)	91	44	0.146	93	35	0.867

^a ^Only female patients with primary, unilateral, invasive breast cancer were included. ^b ^According to the TNM classification of Sobin and Wittekind [38]. ^c ^IRS = immunoreactive score according to Remmele and Stegner [35]. ^d ^Fisher's exact test. Significant p values are marked in bold face.

## Discussion

MIF is not only a mediator of acute and chronic inflammatory conditions [1], but also plays a role in cancerogenesis. MIF overexpression has been observed in several human tumours and molecular links between MIF and p53, apoptosis, JAB1/CSN5, and cell cycle regulation [6,7,18,22,23,39] suggest that MIF is important in regulating the balance between cell homeostasis and neoplastic behaviour. Moreover, MIF has angiogenic potential and enhances the expression of proangiogenic CXCL8 and vascular endothelial growth factor (VEGF) [37,40]. Interestingly, MIF exhibits similarities with CXCL8 and shares with this chemokine an ELR-like motif [41]. In fact, MIF acts as a non-cognate ligand of CXCR2 and drives leukocyte recruitment through CXCR2 [5]. Thus, MIF is a cytokine/chemokine and autocrine/paracrine growth factor promoting tumourigenesis.

Only a few studies have addressed the role of MIF in breast cancer. Bando and colleagues noticed MIF overexpression in 93 primary breast cancer tissues with MIF localizing to tumour as well as stromal cells including TAMs [12]. Of note, in that cohort intra-tumoural MIF levels and circulating MIF inversely correlated with nodal status. Intra-tumoural MIF levels correlated with proinflammatory macrophage cytokines, suggesting that MIF regulates and is regulated by tumour-stroma interactions, particularly in cancers with minimal nodal spread [12]. While the overexpression of MIF in primary human breast cancer tissue was recently confirmed [31], analysis of that cohort (85 patients with MIF-positive and 36 patients with MIF-negative tumours) revealed that positive MIF expression was associated with unfavourable disease-free, but not overall, survival. This latter study also demonstrated that MIF levels in breast tumour tissue correlated with tumour CXCL8 levels, whereas no correlations with steroid hormone receptor status were observed [31]. Thus, while there is consensus from these reports that MIF is overexpressed in human breast cancer, its functional correlation with breast tumourigenesis has remained unclear. In own prior work leading up to the current investigation, we observed strong MIF expression in MCF-7 and ZR-75-1 breast cancer cells, and blockade of MIF secreted from these cells suggested a causal role of extracellular MIF in breast cancer cell survival involving the AKT/PI3K pathway [21]. These findings are in favour of a pro-tumourigenic role of MIF. Similarly, Hagemann *et al. *demonstrated that MIF enhanced MCF-7 invasiveness and matrix metalloproteinase-9 (MMP-9) activity. When coculturing MCF-7 cells with human macrophages, MIF was identified as a prominent target in tumour cells that was upregulated upon inflammatory cytokine production by cocultured macrophages. In turn, induced tumour cell-derived MIF was critical for invasiveness of tumour cells and MMP9 secretion by macrophages [32]. In conjunction, these prior and in part conflicting studies indicate that despite its established overexpression in breast cancer, the contribution of MIF to breast cancerogenesis is likely to be complex and may vary between disease stages. The precise function of MIF may depend on its cellular expression (intracellular, extracellular, breast or stromal cell-derived).

To address these possibilities and to comprehensively explore MIF's role in breast cancerogenesis, we correlated MIF expression levels with clinicopathological data in a large cohort of patients with invasive breast cancer and studied the effect of MIF on the behaviour of breast cancer cell lines *in vitro*. While we confirmed that MIF was markedly overexpressed in non-invasive MDA-MB-468 and ZR-75-1 breast cancer cells, compared to benign MCF-12A breast cells, the highly invasive MDA-MB-231 cancer cells surprisingly showed low MIF expression levels. In contrast, expression of the MIF receptor CD74 was elevated in MDA-MB-231. This suggested that invasive breast cancer cells are target cells of MIF in breast cancer. Indeed, exogenous MIF stimulated MDA-MB-231 proliferation, and blockade of CD74 blocked MDA-MB-231 proliferation induced by autocrine MIF activity. However, enhanced CD74-dependent tumour cell proliferation by autocrine MIF was also measured in MDA-MB-468 cells. These cells also expressed some surface CD74, an observation that explained their responsiveness, but contained markedly reduced levels of total cellular CD74. Only a small portion of CD74 is presented at the cell surface at any given time point, whereas the majority is localized in the endolysosomal compartment. It will be of interest to study, whether signalling from MIF/CD74 complexes originates from the cell surface or is activated from signalling endosomes. In line with our results, secretion of MIF by tumour cells and autocrine stimulation of neoplastic behaviour by MIF has previously been observed for several tumour cell types [14,32,42].

MIF is expressed in numerous cell types including tumour cells. Preformed MIF protein resides in the cytosol, from where it is secreted by a non-conventional pathway upon stimulation. Stimuli such as endotoxin, inflammatory cytokines, or oxidized lipids have all been demonstrated to be potent triggers of MIF secretion [1,43]. Autocrine MIF activation loops have been implicated in tumour cell growth. Here we tested whether exogenous MIF would induce MIF secretion. Both MDA-MB-231 and MDA-MB-468 breast cancer cells slowly secreted MIF during unstimulated cultivation. Unspecific cell death could be excluded (data not shown). Stimulation of cells rMIF led to a dramatic upregulation of the secretion rate of endogenous MIF of up to 500–1000 ng/ml in MDA-MB-468 and MDA-MB-231. Peak secretion occurred after 20 min, indicating that exogenous MIF triggered massive release of preformed MIF stores. Relative upregulation rates over background secretion and added rMIF were even more striking. MIF secretion in invasive MDA-MB-231 cells increased 28-fold after 10 min and reached a sustained rate of 50-fold thereafter. It is worthwhile of mentioning that the concentration of rMIF found to trigger optimal release of endogenous MIF was 150 ng/ml. Although this rMIF concentration is well within the range of MIF concentrations measured and known in pathophysiologic conditions to promote inflammation and pro-tumourigenic behaviour, it is currently mechanistically unclear why lower concentrations of rMIF (10–50 ng/ml) sufficed to lead to an enhancement of breast cancer cell proliferation and invasion. Thus, invasive breast cancer cells exhibiting low MIF expression and secretion levels at baseline are capable of dramatically upregulating MIF upon short term triggering with exogenous MIF, possibly derived from tumour/stroma interactions.

We found that anti-MIF and anti-CD74 antibodies potently blocked breast cancer cell proliferation induced by autocrine or exogenous MIF. Thus, as observed in prostate and gastric cancer [25,26], MIF/CD74 interactions appear to play a role in breast tumourigenesis. It is interesting to note, that upon MIF binding CD74 associates with CD44 and signalling induced by MIF/CD74/CD44 has been implicated in increased B cell survival [28]. The role of the various known CD44 variants in breast cancer is a matter of debate [29]. Recent reports indicate that CD44 overexpression could to be associated with an increased disease-free survival of patients suffering from node-negative invasive breast carcinomas [30,44]. However, numerous other studies have suggested that CD44 promotes invasiveness of breast cancer cells [45-47]. Thus, future studies should address the role of CD74/CD44 complexes in MDA-MB-231 invasion.

In conjunction with the study by Hagemann *et al.*, it may be speculated that in human breast cancer tissue, TAM-derived MIF triggers breast cancer cell activation. In fact, the assumption that MIF promotes breast tumour cell invasiveness was underscored by Matrigel invasion assays using MDA-MB-231. The transmigration rate of these cells through Matrigel was markedly enhanced when rMIF was added to the lower chamber, in line with recent observations demonstrating that MIF functions as a chemoattractant [5,48]. Furthermore, the tremendous increase in the rate of MIF secretion as observed in breast cancer cells following stimulation with rMIF suggests that TAM-derived MIF could trigger MIF secretion from breast cancer cells, thus igniting a local MIF amplification loop. In turn, as manifest from the study of Hagemann [32], breast cancer cell-derived MIF might then modulate (pro-invasive) TAM activities as well as induce a broad range of inflammatory processes, including the elevated expression of secondary mediators that may further promote tumourigenesis [14,22].

As MIF is expressed in breast cancer cell lines, promotes breast tumour cell proliferation and invasion, and contributes to stroma/tumour interactions, one may expect that MIF is overexpressed in breast cancer *in vivo *and would correlate with a poor survival prognosis and markers such as EGFR or HER2 [49].

We compared intra-tumoural MIF levels in specimens from 175 breast cancer patients with those of 35 normal breast tissues. Confirming earlier studies by Bando *et al. *[12] and Xu and colleagues [31], we found that MIF protein was significantly upregulated in breast cancer tissues compared to normal breast epithelium. However, there are differences between our study and the two previous investigations. We detected substantial but varying concentrations of MIF protein in essentially all tumour cells, where MIF was primarily localised in the cytosol. In contrast, Bando and colleagues in their cohort of 93 primary cancer tissues predominantly observed nuclear MIF staining. In the cohort studied by Xu et al., surprisingly only 1/3 of the patients (n = 36) had MIF-positive breast cancer tissues. Xu et al. used a polyclonal anti-MIF antibody, whereas in our study the highly specific anti-MIF MAB 289 was applied.

At first sight, our *in vitro *data are in contrast to the correlations observed *in vivo*. We noticed a significant positive correlation between MIF levels and hormone receptors (especially PR) and a significant negative correlation between MIF and tumour size. PR and ER are markers of a favourable prognosis in breast cancer and characterize specific subtypes of the disease. Xu *et al. *did not detect any correlation between MIF and ER or PR in their cohort, but picked up a positive correlation with EGFR, with which we also see a moderately significant correlation (p = 0.038). Bando *et al. *only measured ER and found no correlation with MIF. Despite these differences that may in part be due to the different cohorts studied, the link between MIF levels and ER/PR status as observed in our cohort is of interest, because these parameters may be molecularly connected to MIF through JAB1/CSN5, which is abundantly expressed in breast cancer [50]. JAB1 is an intracellular binding partner of MIF [7] and has been demonstrated to interact with ER and PR [51].

All three studies concur in failing to see any correlations between breast cancer MIF expression levels and tumour size or histological tumour grades [12,31]. Interestingly, Bando *et al. *noticed that MIF levels in breast tumour tissue were *inversely *correlated with nodal status. However, no such correlation was detected in our study and in the study by Xu *et al.*. The observation that abundant MIF expression is significantly associated with breast tumours of small size supports the notion that intracellular MIF can inhibit tumour cell proliferation. This hypothesis is underscored by the finding that breast cancer patients with abundant MIF expression have a favourable prognosis both according to tumour-specific OS and RFS. The strong statistical significance of these data suggests that high levels of MIF expressed in the cytosol of breast carcinoma cells are beneficial for the outcome of breast cancer.

Thus, there appears to be a dichotomy of MIF functions in breast cancer progression. We speculate that intracellular MIF in the breast cells has a protective function, whereas extracellular MIF, be it TAM-derived or produced by carcinoma cells upon stroma/tumour interactions, is pathogenic. The anti-tumour effect of cytosolic breast epithelial MIF might be mediated through JAB1/CSN5 which promotes p27 degradation [52] and is counter-regulated by MIF [7] or could reflect cell homeostatic activities of MIF [23,53]. Pro-tumourigenic effects of extracellular MIF have been reported [31,32] and could be due to MMP activation, or MIF's pro-angiogenic and inflammatory activity. In fact, it was suggested that MIF is a determinant of the M1-subtype of TAMs and that mammary adenocarcinoma cells lead to MIF ablation in M1-TAMs, inducing a switch towards M2 polarization [54].

## Conclusion

This study in conjunction with prior observations by others indicates that MIF has a dual role in breast cancer. Intracellular MIF localised to breast cancer cells may be indicative of a favourable prognosis, whereas extracellular breast tumour tissue-derived MIF could be proinflammatory and will likely constitute an unfavourable prognosis marker.

## List of abbreviations

ER: estrogen receptor; EGFR: epidermal growth factor receptor; IRS: immunoreactive score; JAB1/CSN5: c-Jun activation domain binding protein-1/COP 9 signalosome subunit 5; MIF: macrophage migration inhibitory factor; MMP: matrix metalloproteinase; OS: overall survival; PR: progesterone receptor; RFS: recurrence-free survival; TAM: tumour-associated macrophage; TMA: tissue microarray.

## Competing interests

J.B. is an inventor on patent applications on anti-MIF strategies. The other authors declare that they have no competing interests.

## Authors' contributions

EV and EN carried out most of the experiments, participated in data analysis, and assisted in drafting the manuscript. NB participated in TMA analysis; AS, HL, and BL participated in the in vitro experiments. AH provided the TMA and participated in data analysis. ED conceived the TMA part of the study, participated in experiment design and data analysis, wrote parts of the manuscript, and revised the manuscript. JB conceived the in vitro part of the study as well as the overall study, participated in experiment design and data analysis, and wrote and revised the manuscript. All authors read and approved the final manuscript.

## Pre-publication history

The pre-publication history for this paper can be accessed here:

http://www.biomedcentral.com/1471-2407/9/230/prepub

## Supplementary Material

Additional file 1 **Clinicopathological parameters of breast cancer specimens**. The table shows clinicopathological parameters of 175 breast cancer specimens analysed on a tissue microarray. Click here for file
