# Supplementary material for: Dual role of macrophage migration inhibitory factor (MIF) in human breast cancer
Source: BMC Cancer. 2009 Jul 14;9:230. doi: 10.1186/1471-2407-9-230 (PMC2716369; doi:10.1186/1471-2407-9-230)
Supplement: Additional file 1 — Clinicopathological parameters of breast cancer specimens. The table shows clinicopathological parameters of 175 breast cancer specimens analysed on a tissue microarray. [file 1471-2407-9-230-S1.doc]

| **Clinicopathological parameters of 175 breast cancer specimens analysed on a tissue microarray** | | | |
| --- | --- | --- | --- |
| **Variable** | **Categorisation** | ***n^a^*analysable** | **%** |
| **Clinicopathologic parameters** |  |  |  |
| Age at diagnosis | median 57.5 years  (range 25-82 years) |  |  |
|  | <50 years | 51 | 29.1 |
|  | ≥50 years | 124 | 70.9 |
| Tumour size^c^ |  |  |  |
|  | pT1 | 48 | 27.4 |
|  | pT2 | 86 | 49.1 |
|  | pT3 | 12 | 6.9 |
|  | pT4 | 27 | 15.4 |
|  | pT x^b^ | 2 | 1.2 |
| Lymph node status^c^ |  |  |  |
|  | pN0 | 67 | 38.3 |
|  | pN1-3 | 100 | 57.1 |
|  | pN x | 8 | 4.6 |
| Histological grade |  |  |  |
|  | G1 | 19 | 10.9 |
|  | G2 | 73 | 41.7 |
|  | G3 | 79 | 45.1 |
|  | G x | 4 | 2.3 |
| Histological type |  |  |  |
|  | invasive ductal | 145 | 82.9 |
|  | Invasive lobular | 13 | 7.4 |
|  | other | 17 | 9.7 |
| Estrogen receptor status |  |  |  |
|  | negative (IRS^d^ 0-2) | 46 | 26.3 |
|  | positive (IRS 3-12) | 96 | 54.9 |
|  | IRS x | 33 | 18.9 |
| Progesterone receptor status |  |  |  |
|  | negative (IRS 0-2) | 101 | 57.7 |
|  | positive (IRS 3-12) | 49 | 28 |
|  | IRS x | 25 | 14.3 |
| EGFR status |  |  |  |
|  | negative (IRS 0) | 65 | 37.1 |
|  | positive (IRS 1-3) | 92 | 52.6 |
|  | IRS x | 18 | 10.3 |
| ^a^Only female patients with primary, unilateral, invasive breast cancer were  included in the study. ^b^x status unknown. ^c^According to the TNM classification by Sobin and Wittekind (Sobin and Wittekind, 1997). ^d^IRS= Immunoreactive score according to Remmele and Stegner (Remmele and Stegner, 1987). | | | |
